# Opportunistic detection of atrial fibrillation using blood pressure monitors: a systematic review

**DOI:** 10.1136/openhrt-2015-000362

**Published:** 2016-04-12

**Authors:** Sarah A Kane, James R Blake, Frank J McArdle, Philip Langley, Andrew J Sims

**Affiliations:** 1 Newcastle upon Tyne Hospitals NHS Foundation Trust, Royal Victoria Infirmary, Newcastle upon Tyne, UK; 2 Institute of Cellular Medicine, Newcastle University, Newcastle upon Tyne, UK; 3 School of Engineering, University of Hull, Hull, UK

**Keywords:** Systematic Review, Opportunistic Screening

## Abstract

**Background:**

Atrial fibrillation (AF) affects around 2% of the population and early detection is beneficial, allowing patients to begin potentially life-saving anticoagulant therapies. Blood pressure (BP) monitors may offer an opportunity to screen for AF.

**Aim:**

To identify and appraise studies which report the diagnostic accuracy of automated BP monitors used for opportunistic AF detection.

**Methods:**

A systematic search was performed of the MEDLINE, MEDLINE In-Process and EMBASE literature databases. Papers were eligible if they described primary studies of the evaluation of a BP device for AF detection, were published in a peer-reviewed journal and reported values for the sensitivity and specificity. Included studies were appraised using the QUADAS-2 tool to assess their risk of bias and applicability to opportunistic AF detection. Values for the sensitivity and specificity of AF detection were extracted from each paper and compared.

**Results and Conclusions:**

We identified seven papers evaluating six devices from two manufacturers. Only one study scored low risk in all of the QUADAS-2 domains. All studies reported specificity >85% and 6 reported sensitivity >90%. The studies showed that BP devices with embedded algorithms for detecting arrhythmias show promise as screening tools for AF, comparing favourably with manual pulse palpation. But the studies used different methodologies and many were subject to potential bias. More studies are needed to more precisely define the sensitivity and specificity of opportunistic screening for AF during BP measurement before its clinical utility in the population of interest can be assessed fully.

## Introduction

Atrial fibrillation (AF) is a common cardiac arrhythmia that affects around 2% of the population and around 8% of those aged over 75 years.^1^ The presence of AF can increase a patient's risk of stroke by up to a factor of five;^2^ however, this risk can be significantly reduced through anticoagulation therapy for those considered at medium or high risk according to the CHA_2_DS_2_-VASc scoring system,^3^ making the early detection of AF beneficial. In the UK, opportunistic screening for AF has been shown to be cost-effective.^4^

Routine measurement of blood pressure (BP) is one opportunity for detecting AF. In the UK, the evidence-based National Institute for Health and Care Excellence (NICE) has published guidance on the clinical management of hypertension.^5^ This guidance recommends pulse palpation before taking a BP measurement as some automated sphygmomanometers are known to be inaccurate for patients with an irregular pulse. Some manufacturers have turned this problem into an advantage and have introduced automated sphygmomanometers that incorporate algorithms for detecting AF. In 2013 NICE published a medical technology guidance which concluded that the use of these devices could be beneficial in primary care.^6^ ^7^

In this paper, we aim to identify all primary studies that have evaluated the use of automated sphygmomanometers for the detection of AF against a reference standard, to appraise these studies based on their relevance to opportunistic AF detection and to compare their results for the sensitivity and specificity of AF detection.

## Methods

A systematic search was performed in August 2014 of the MEDLINE, MEDLINE In-Process and EMBASE literature databases using the keywords ‘blood pressure device’ and ‘atrial fibrillation screening’ and subject headings ‘blood pressure determination’, ‘blood pressure monitoring, ambulatory’, ‘sphygmomanometers’, ‘atrial fibrillation’ and ‘cardiac arrhythmias’. The search was restricted to English language, non-animal studies published between January 1990 and August 2014. Papers were eligible if they described a primary evaluation of at least one BP device for the detection of AF, were published in a peer-reviewed journal and reported values for the sensitivity and specificity of AF detection in the population studied. One person (SAK) screened the identified titles and abstracts for eligible studies. Studies that fulfilled all of the eligibility criteria and those which could not be ruled out on the basis of title and abstract were retrieved in full-text form. These were further reviewed until only studies that fulfilled the eligibility criteria remained.

The study characteristics and reported values for the sensitivity and specificity of AF detection, compared with a reference standard, were extracted from each paper. We appraised the studies using the QUADAS-2 tool^8^ for their risk of bias and applicability to the clinical problem (ie, detection of AF during opportunistic screening) against four domains: patient selection, index test, reference standard and flow and timing. For each paper, the contingency tables were reconstructed. To ensure consistency, CIs for sensitivity and specificity were estimated using a binomial approximation as described by Clopper and Pearson.^9^

## Results

### Search results

**Figure 1:**
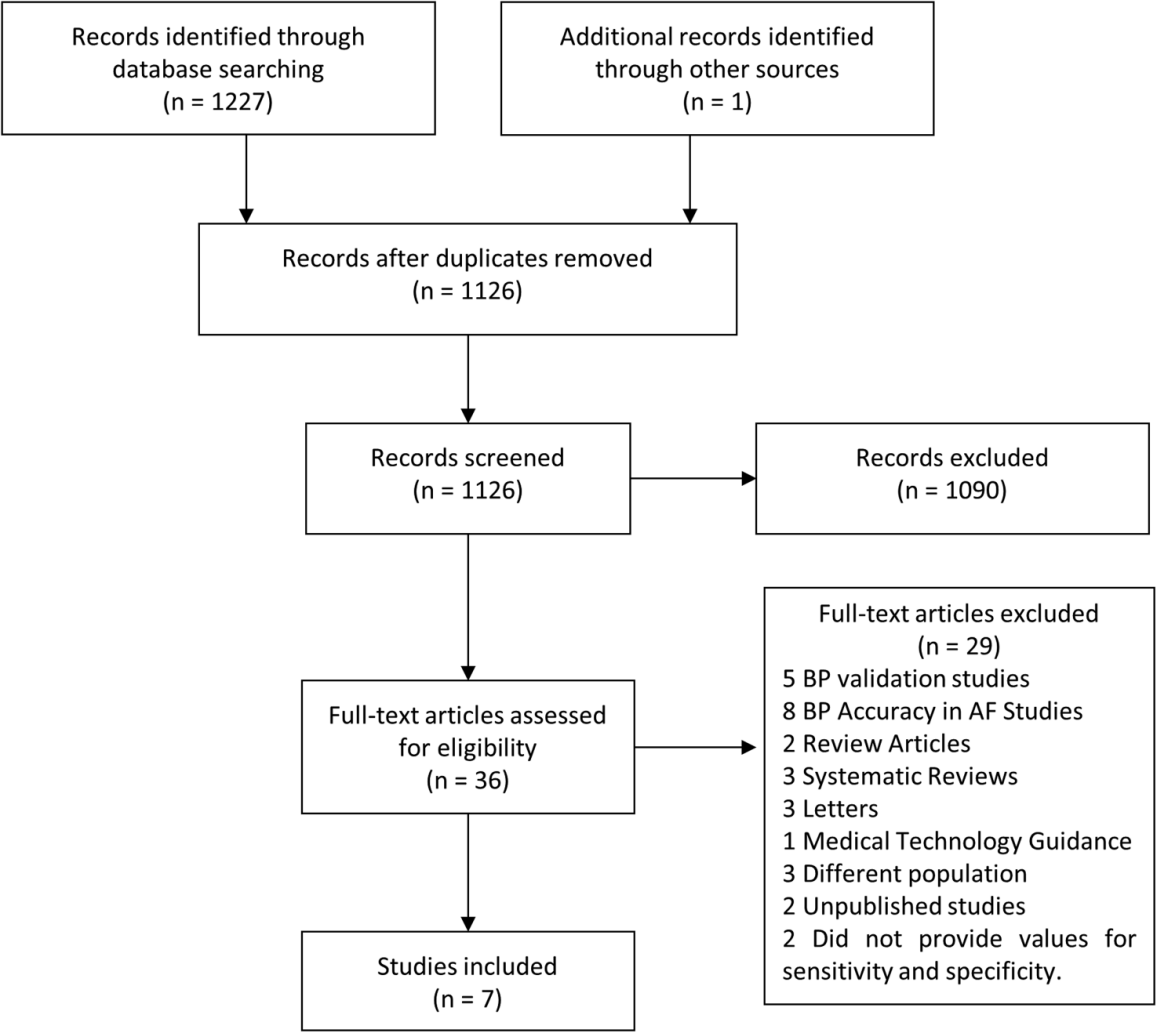
PRISMA diagram showing the results from each stage of the search process. PRISMA, Preferred Reporting Items for Systematic Review and Meta-Analyses.

Figure 1 shows the Preferred Reporting Items for Systematic Review and Meta-Analyses (PRISMA) flow chart depicting the search results at each stage.^10^ We identified seven papers describing primary studies.^11–17^ Of these, six were found during the main search; one further paper was published after the search date and was identified by a journal alert using the same search criteria.^17^ The studies included six devices from two manufacturers. Two of the papers analysed more than one device (table 1).

**Table 1:** Summary of study characteristics for each of the included studies

				Population			
Paper	Device studied	Reference standard	Number of measurements per participant	Number of participants	Male (%)	Mean age (years)	AF prevalence (%)
Wiesel *et al*^11^	Omron 712C	12-lead ECG	2	450	59	69	12.0
Stergiou *et al*^12^	Microlife BPA100 Plus	12-lead ECG+1-lead ECG	3	73	66	71	37.0
Wiesel *et al*^13^	Microlife BPM BP3MQ1–2D	12-lead ECG	3	405	51	73	23.0
Marazzi *et al* (a)^14^	Microlife BP A200 Plus	12-lead ECG	1	503	54	67	20.1
Marazzi *et al* (b)^14^	Omron M6	12-lead ECG	1	503	54	67	20.1
Wiesel *et al*^15^	Microlife BPM BP3MQ1–2D	ECG event monitor	1–4 per day	139	37	67	12.9
Kearley *et al*^16^	WatchBP	12-lead ECG	1	999	49	80	7.9
Wiesel *et al* (a)^17^	Microlife BP A200 Plus	12-lead ECG	3	183	59	74	16.4
Wiesel *et al*^17^ (b)	Omron M6	12-lead ECG	1	183	59	74	16.4

AF, atrial fibrillation.

### Appraisal of risk of bias and study applicability

The seven studies were appraised using the QUADAS-2 tool^8^ (table 2) which considers four different domains (patient selection, index test, reference standard and flow and timing). Each of the four domains is appraised in terms of risk of bias and for the first three the applicability of the study to the review question is also considered. One of the eligible studies scored low risk over all four domains.^16^

**Table 2:** Summary of the results from the QUADAS-2 tool for each of the included papers

	Risk of bias				Applicability concerns		
Study	Patient selection	Index test	Reference standard	Flow and timing	Patient selection	Index test	Reference standard
Wiesel *et al*^11^	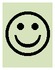	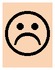	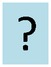	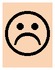	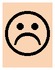	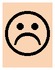	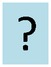
Stergiou *et al*^12^	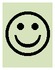	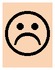	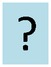	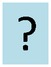	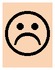	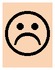	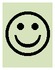
Wiesel *et al*^13^	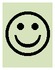	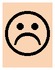	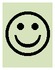	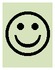	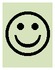	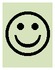	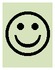
Marazzi *et al*^14^	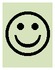	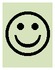	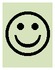	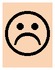	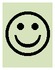	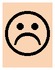	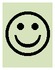
Wiesel *et al*^15^	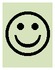	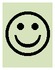	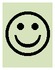	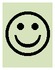	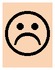	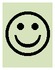	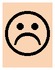
Kearley *et al*^16^	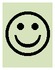	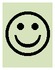	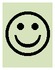	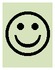	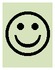	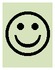	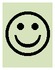
Wiesel *et al*^17^	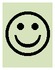	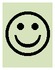	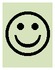	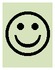	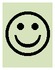	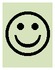	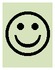

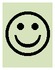
, low risk; 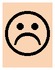, high risk; 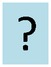, unclear risk.

#### Patient selection

All of the included studies selected patients based on predefined criteria and so were considered to be at a low risk of bias for patient selection.

Three studies were considered to have low applicability to the review question (detection of AF from opportunistic screening). In two of these, the authors studied participants from populations likely to benefit from opportunistic screening,^11^ ^15^ but who also had additional risk factors for AF, which may limit the extent to which their findings can be applied to opportunistic screening. In the remaining study, an experimental case-control design was adopted^12^ rather than enrolling a consecutive or random sample of patients, reducing the applicability to opportunistic screening. For the majority of the studies the objective was to assess the performance of the device in the chosen population, however two were application studies; one considering self-monitoring at home^15^ and the other in general practice.^16^

Of the studies considered highly applicable to opportunistic screening, Kearley *et al*^16^ considered a population most likely to benefit—primary care patients over 75 years—and recruited a large number (n=999) of participants to estimate test sensitivity with a precision better than 10%. Other authors^13^ ^14^ ^17^ considered populations also likely to benefit from opportunistic screening but with a higher prevalence of AF, such as cardiology or hypertension clinic outpatients.

#### Index test

Three of the included studies were considered at high risk of bias.^11–13^ In these studies the authors calculated the sensitivity and specificity of AF detection using all measurements from all patients, with the assumption that each measurement was independent. However repeated measurements within individuals are likely to be correlated, potentially breaking the assumption of independence and leading to underestimates of measurement uncertainty.

Three of the seven included studies used a valid index test (automated BP monitor with embedded AF detection algorithm) and so were considered to have high applicability to the review question.^13^ ^15^ ^16^ Three of the seven studies used devices which claim to detect irregular heartbeat or arrhythmias but make no claims about AF detection in particular (Omron M6 and Microlife BP A100 plus).^12^ ^14^ ^17^ The remaining study used a modified device and the collected data were downloaded onto a laptop for analysis.^11^

Throughout all of the included studies, there was a large degree of variation in the methods used for interpreting the index test. Two of the papers^14^ ^16^ made a diagnosis of AF on the basis of a single BP measurement. The remainder of the studies took multiple measurements from each participant and assessed the impact of different interpretations on the sensitivity and specificity. Four of the studies considered individual measurements and majority rule methods.^11–13^ ^17^ The remaining study,^15^ carried out in the home environment over 30 days with between one and four measurements per day, considered two methods: using the first reading of the day only and using an overall daily AF status based on the repeat measurements.

#### Reference standard

The reference standard for AF diagnosis is a 12-lead ECG interpreted by a certified cardiologist.^18^ Of the seven papers examined, four performed a 12-lead ECG prior to or shortly after the BP measurement that was interpreted by at least one cardiologist blinded to the result of the index test.^13^ ^14^ ^16^ ^17^ These studies were considered to have a low risk of bias and high applicability to the review question (likely to correctly classify AF).

The three remaining studies were either unclear about their use of a reference standard or adopted different approaches. Stergiou *et al*^12^ made the diagnosis of AF on the basis of a 12-lead ECG recording interpreted by a cardiologist; however, no blinding was mentioned (questionable risk of bias). Wiesel *et al*^11^ did not state the number of leads used for the ECG nor how the ECG was interpreted, so the risk of bias and applicability were classified as questionable. The remaining study,^15^ which was the only one to assess a device in the home environment, used an ECG event monitor that was used by the participant to obtain a 60 s recording prior to each BP reading. This method is less likely to correctly diagnose AF (low applicability); however, the results were interpreted by a blinded cardiologist (low risk of bias).

#### Flow and timing

Two main sources of bias regarding the flow and timing within the included studies were considered: the time interval between the reference standard and the index test and inappropriate inclusions/exclusions of participants following enrolment. Studies were considered to have a high risk of bias (table 2) if they did not fulfil the criteria for either category.

As AF can be paroxysmal, the ECG and BP measurements should be performed simultaneously to ensure the validity of the reference test. However, given the practical considerations, for this appraisal, the time interval was considered adequate if the ECG and BP measurements were performed within the same clinic visit. Marazzi *et al*^14^ was the only study to record 12-lead ECG and BP measurements simultaneously. Stergiou *et al*^12^ performed a baseline 12-lead ECG prior to the measurement and then single-lead ECG during the BP measurement. The remainder of the studies performed the reference test during the same clinic visit (and all within 30 min of the BP measurements) and so all included studies were considered at low risk of bias for this category.

Three studies were considered to have high or questionable risk of bias for inappropriate inclusions/exclusions of participants (table 2). Marazzi *et al*^14^ excluded enrolled patients who had >5 mm Hg difference in BP when measured in both arms. In this case, the measurement uncertainties may be greater than reported as participants in which measurement was difficult have been excluded. Wiesel *et al*^11^ included patients returning for repeat tests in the analysis multiple times without taking into account repeated-measures effects. Both of these papers were therefore classed as having an increased risk of bias. The paper by Stergiou *et al*^12^ was considered to have a questionable risk of bias as the authors state in the text that two participants only had two BP measurements made instead of three; however, this is not in agreement with the results provided in the corresponding table in the paper where 72 of 73 participants are accounted for. In the 2013 paper by Wiesel *et al*,^15^ several participants did not adhere to the guidelines and so could not be included in the analysis; however, this was beyond the control of the authors and suitable corrections were made when reporting the results, so this study was considered to have a low risk of bias.

### Review of study findings

**Figure 2:**
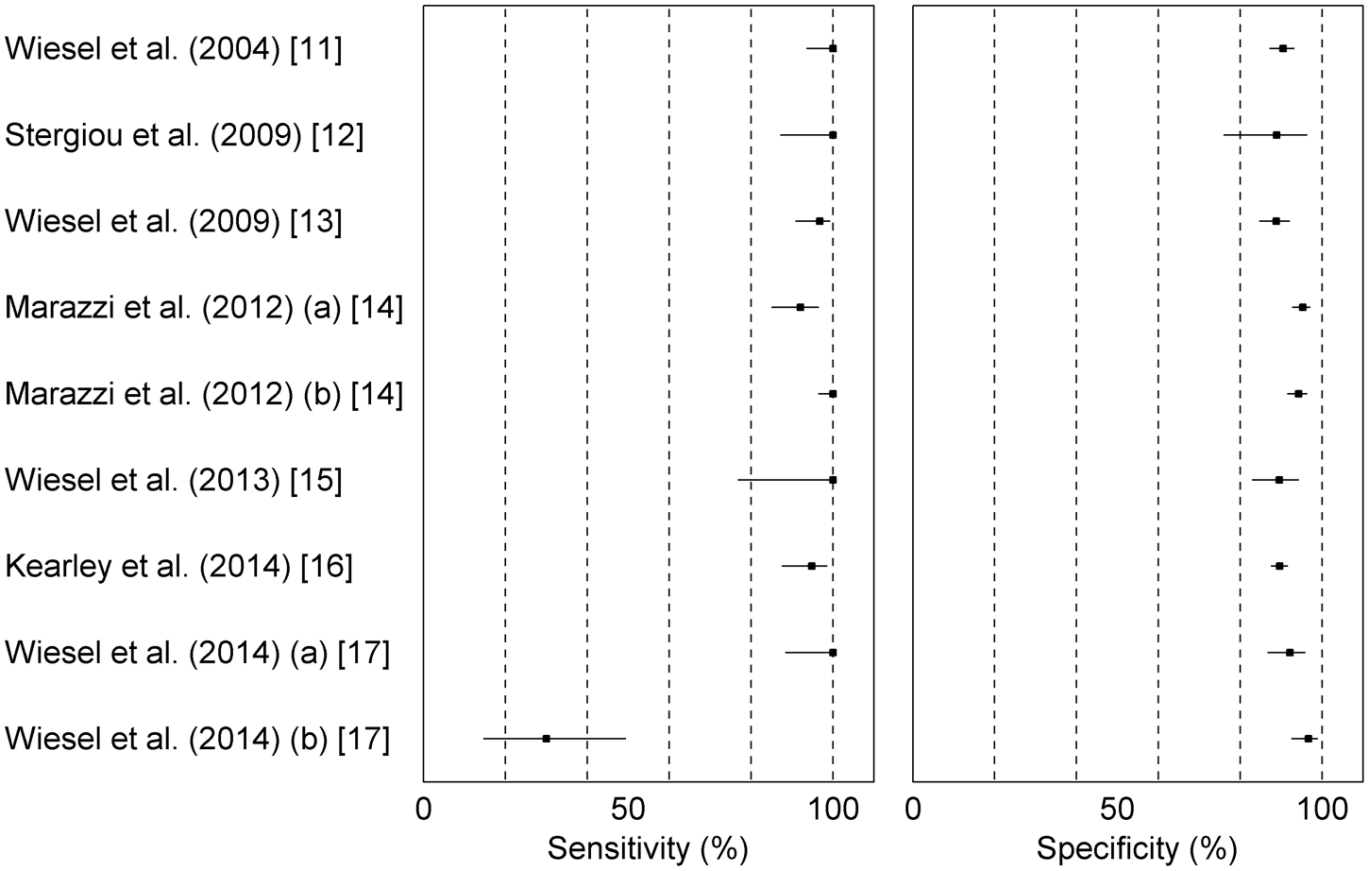
Sensitivity and specificity of AF detection for each of the included studies. CIs have been calculated using a binomial approximation as described by Clopper and Pearson.^9^

The extracted results for the sensitivity and specificity are shown in figure 2. For the studies where multiple methods for assessing the index test were investigated, the best reported values are shown. In one study^14^ a discrepancy relating to the specificity of the Microlife BP A200 was noted between the result quoted in the text and the value calculated from the results table; we used the latter. The studies consistently showed that AF detection specificities >85% can be achieved irrespective of the device used, provided that there is an appropriate method for interpreting the index test. Six of the seven studies reported sensitivity >90%. However, in the remaining paper,^17^ one device demonstrated a much lower sensitivity (30%, Omron M6). This result was in direct conflict with the results obtained by Marazzi *et al*^14^ who found a sensitivity of 100% for the same device while still achieving high specificity. Wiesel *et al* speculate that this difference may be due to the population used by Marazzi *et al*, which had a high prevalence of AF and relatively young age. However, there have been other studies using populations of similar age^11^ and a higher prevalence of AF^12^ ^13^ that showed high sensitivities and specificities, so the discrepancy remains unexplained.

## Discussion

### Summary

Seven papers were found that reported primary studies of AF detection using automated BP devices. The papers tested several methods for interpreting the index test results in a variety of patient populations and only one of the papers scored low risk in all four of the QUADAS-2 domains.^16^ In general, the results were conclusive, with all specificities reported to exceed 85% and most detection sensitivities reported to exceed 90% (one study reported a device sensitivity of 30%).

### Strengths and limitations

This review employed wide search criteria to capture all of the relevant articles following which strict inclusion criteria were applied to identify articles determining the diagnostic accuracy of these devices in particular populations and settings. However, we appreciate that the focus of other studies may have been clinical utility rather than diagnostic accuracy. For example, Ermini *et al*^19^ studied a large population to investigate the effect of using a modified BP device on the number of new cases of AF detected, and Wiesel *et al*^20^ assessed the rate of false positives in a group of 19 participants using the device in a home environment. Additionally, non-peer-reviewed work has been excluded such as the research letter by Huang *et al*,^21^ a small scale study assessing the use of these devices in the detection of cardiac arrhythmias, not specifically AF.

### Comparison with existing literature

A previous review of this topic by Verberk and de Leeuw^22^ focused on how the interpretation of the index test affected diagnostic accuracy. They pooled data to determine mean values for sensitivity and specificity, assuming homogeneity of the studies. However, we found that previous studies had heterogeneous populations and index tests. This is particularly highlighted through the studies by Marazzi *et al*^14^ and Wiesel *et al*,^17^ where two different studies of the same device gave substantially different results. Additionally, our paper highlights the three peer-reviewed studies^15–17^ published since the Verberk and de Leeuw paper, emphasising that this is an active area of research.

A recently published meta-analysis by Taggar *et al*^23^ identifies published methods for detecting AF and includes automated BP devices as well as pulse palpation, smartphone applications and non-12-lead ECG devices. Of the techniques identified, they found BP monitors and non-12-lead ECG devices to have the highest diagnostic accuracy. Their review compared different technologies for AF detection in a range of patients and settings, whereas ours specifically considers diagnostic accuracy studies for opportunistic detection of AF during BP measurement.

A further review and meta-analysis by Verberk *et al*^24^ described evidence and practice recommendations relating to AF detection using blood pressure devices. Their paper focused on a particular family of BP devices which incorporate a specific AF detection algorithm (Microlife AG, Switzerland) and concluded that three sequential measurements should be used, and that a suspected diagnosis be confirmed by ECG. They also proposed that automated at-home or ambulatory BP measurements may increase the chance of diagnosis of patients with paroxysmal or asymptomatic AF. The present review was not restricted to a particular family of devices.

### Implications for research and practice

Eligible studies reported to date show good agreement and high values for both sensitivity and specificity. However, the presence of an outlier raises some concerns about the applicability of the studies to routine practice. There is also a lack of homogeneity between the study designs. Many have been carried out in small patient groups which are not representative of the population of interest or use BP devices which do not claim to be suitable for AF detection. We found only one study of high methodological quality which specifically considered the population most likely to benefit from opportunistic screening,^16^ highlighting the need for further large-scale, well-designed studies. The recently published study protocol by Uittenbogaart *et al*^25^ describes a yearlong randomised control trial that aims to test the use of automated BP devices for opportunistic AF detection (alongside other screening methods) in 96 primary care practices in the Netherlands and will provide further evidence in this area.

BP devices with embedded algorithms show promise as a screening tool for AF. The high sensitivities and specificities reported for these devices compare favourably with the current screening practice of manual pulse palpation which has the sensitivity and specificity of 94% and 72%, respectively.^26^ The introduction of these devices into routine practice could provide a simple, objective method for AF screening in high-risk groups.
